# Outcomes and predictors of tuberculosis mortality in Kweneng West District, Botswana: a retrospective cohort study

**DOI:** 10.11604/pamj.2022.42.1.32381

**Published:** 2022-05-02

**Authors:** Keatlaretse Siamisang, Goabaone Rankgoane-Pono, Tumisang Malebo Madisa, Tantamika Mudiayi, John Thato Tlhakanelo

**Affiliations:** 1 Department of Family Medicine and Public Health, University of Botswana, Gaborone, Botswana,; 2 Department of Health Services Management, Ministry of Health and Wellness, Gaborone, Botswana

**Keywords:** Outcomes, predictors, tuberculosis, mortality, Botswana

## Abstract

**Introduction:**

Botswana is among the countries with the highest tuberculosis (TB) notification rates in the world. However, there is paucity of data on the outcomes and predictors of TB mortality at district level in Botswana. This study was aimed at describing the TB outcomes and identifying the predictors of mortality in Kweneng West district, Botswana.

**Methods:**

this was a retrospective cohort study of TB outcomes in Kweneng West, from January 2008 to December 2016. All documented drug-sensitive TB (DS-TB) patients aged 16 years and above were included. The World Health Organization (WHO) definitions of treatment outcomes for DS-TB were used. Binary logistic regression was used to identify predictors of mortality.

**Results:**

there were 1475 TB notifications in the study period. The median age was 36 years and 41.5% were female. A total of 728 (49.4%) were HIV positive. Pulmonary TB (PTB) accounted for 87.3% of all cases. The overall treatment success rate (TSR) was 81.9% and the mortality rate was 9.4%. Compared to the 16-25 years age group, patients aged more than 65 years had the highest risk of mortality (AOR=9.63). Other significant predictors of mortality were male sex (AOR=1.63), no sputum microscopy (AOR=1.77), positive HIV (AOR=2.13) and unknown HIV status (AOR=4.47). Positive sputum microscopy (AOR=0.50) and extra-pulmonary TB (EPTB) (AOR=0.56) were associated with less mortality.

**Conclusion:**

while Botswana has relatively good TB treatment success rates, the mortality rates are high. Public health interventions should target the identified risk factors of mortality.

## Introduction

Tuberculosis (TB) is one of the most common causes of death and is ahead of HIV as the leading cause of death from a single infectious agent [1, 2]. This is despite the disease being curable and preventable [2]. More than 80% of drug-sensitive TB (DS-TB) patients can be successfully treated with a 6-month treatment regimen [1]. TB treatment is said to have prevented about 60 million deaths since 2000 [2]. However, TB continues to have a high morbidity and mortality, especially in resource-limited countries [2].

The World Health Organization (WHO) and the United Nations (UN) have committed to ending the TB pandemic through effective implementation of the end TB strategy and the attainment of the Sustainable Development Goals (SDGs) [2]. The WHO Africa region has borne the brunt of the TB pandemic. Despite being home to 10% of the world´s population, the region accounts for 25-30% of all TB cases [2, 3]. Furthermore, 17 of the 30 countries with the highest TB burden are in the continent [4]. The 30 high burden countries are said to account for 90% of all annual TB morbidity [1]. Within Africa, TB is mainly fuelled by multiple social determinants of health, including poverty and political and economic instability that hinder the development or sustainability of robust health systems [1]. Furthermore, often TB disease is associated with precarity, i.e.: vulnerability, marginalization, stigma and discrimination of affected populations [2].

Botswana is a Southern African country with a high burden of TB. The TB -HIV coinfection rate in the country is about 60% [5]. This poses a complex public health challenge as HIV increases the incidence of active TB and TB is associated with progression of HIV [6]. TB reporting in Botswana is organized such that health facilities report TB cases to their district TB coordinators, who in turn report to the Botswana National TB Program (BNTP) at the Ministry of Health and Wellness (MoHw). In addition to TB registers, TB data is also captured on an electronic system called Open Medical Record System (OpenMRS). TB management is free of charge in the country and is administered through Directly Observed Therapy (DOT) at facility level and Community based DOT.

Despite a high burden of TB in Botswana and existence of adequate logistics for programmatic management, there is paucity of data on TB outcomes and predictors of mortality at district level in Botswana. Kweneng district is a rural district in South Western Botswana with a high burden of TB. A study in 2010 revealed relatively low cure and success rates in the district [7]. This study was therefore aimed at describing the TB outcomes and identifying the predictors of TB mortality in Kweneng West district, Botswana. The findings of this study are expected to guide TB public health interventions in the country.

## Methods

**Study design:** this was a retrospective cohort study of the TB outcomes in Kweneng West health district, Botswana, over a 9-year period from January 2008 to December 2016. All patients with drug-sensitive TB notified in the district were retrospectively followed until their outcome. This data is routinely collected on an electronic records system. A cohort study is the most appropriate approach, since it allows inclusion of participants based on exposure status. In this case, the exposure status was having drug sensitive TB. The outcomes were mortality. The outcomes were as detailed in the data collection section below.

**Setting:** Botswana is among the countries with the highest TB notification rates in the world [8]. The study utilized programmatic data from Kweneng West, one of the 27 health districts in Botswana. Kweneng West health district is a mainly rural area located about 100 kilometres from the capital city, Gaborone. It has a population of 47,797 according to the 2011 national census [9]. The district headquarters are in Letlhakeng village. The HIV prevalence at the last national survey in 2013 was 11.8% [9]. The district has one military airbase hospital, 8 clinics, 16 health posts and 31 mobile stops. Most people in the district have reliable access to TB treatment through one of these facilities.

### Study population

***Inclusion criteria:*** all documented DS-TB patients aged 16 years and above and patients notified in Kweneng West health district.

***Exclusion criteria:*** patients under 16 years of age; patients notified in other districts and drug-resistant TB patients.

**Data collection:** Kweneng West health district patient level data was collected from the BNTP records. The national TB data for all districts is held in the Open Medical Records System (Open MRS). Open MRS is a digital data-capturing platform for TB data. The data extracted included patient age, sex, HIV status, TB classification (site of infection), disease category (new/retreatment), microscopy results and TB outcome. The World Health Organization (WHO) definitions of treatment outcomes for DS-TB described below were used in the collection and reporting of TB data [10]:

***Cured:*** a pulmonary TB patient with bacteriologically confirmed TB at the beginning of treatment who was smear or culture negative in the last month of treatment and on at least 1 previous occasion.

***Treatment completed:*** a TB patient who completed treatment without evidence of failure BUT with no record to show that sputum smear or culture results in the last month of treatment and on at least one previous occasion were negative, either because tests were not done or results are not available.

***Treatment failed:*** a TB patient whose sputum smear or culture is positive at month 5 or later during treatment.

***Died:*** a TB patient who dies for any reason before starting or during the course of treatment.

***Lost to follow up:*** a TB patient who did not start treatment or whose treatment was interrupted for two consecutive months or more.

***Not evaluated:*** a TB patient for whom no treatment outcome is assigned.

**Treatment success:** the sum of cured and treatment completed.

**Data analysis:** the data from open MRS was converted into a Microsoft Excel format. After data cleaning and preparation, Statistical Package for Social Sciences (IBM SPSS) version 27, 2020 software was used for data analysis. Categorical data was summarized with frequencies and percentages, while numeric data was summarized with medians and interquartile ranges. Binary logistic regression was used to identify predictors of mortality among the TB patients. A p value of <0.05 was considered to signify significant association between the predictor and outcome variables in the bivariate and multivariate regression models. The dependent variable was mortality, while the independent variables were demographic, clinical and some laboratory variables.

**Ethical considerations:** this analysis only utilized routinely collected data and did not involve any interactions with the patients. All patient identifiers were removed from the data after data extraction. Furthermore, access to the data was limited to authorized personnel. Ethics approval for the study was sought and obtained from the University of Botswana office of research and development (ORD) and the MoHw ethics review board.

## Results

From 2008 to 2016, there were, 1475 DS-TB notifications in Kweneng West health district. Table 1 shows the baseline characteristics of these patients. The median age was 36 years (IQR 27-49) and 41.5% of them were female. Of all the reported cases, 1220 (82.7%) were new notifications while 225 (15.3%) were relapse patients. The rest (2%) were retreated cases having previously failed treatment or lost to follow up. Pulmonary TB (PTB) accounted for 87.3% of cases, while sputum microscopy was performed in 72% of patients and was positive in 50.5% of them.

**Table 1:** baseline characteristics of TB notification in Kweneng West, Botswana, 2008 to 2016 (N=1475)

Variable	Number	Percentage
**Age (Years)**		
Data available	1475	100.0
Median (IQR)	36 (27-49)	
Age range (years)	17-99	
16-25	279	18.9
26-35	425	28.8
36-45	332	22.5
46-55	177	12.0
56-65	137	9.3
>65	125	8.5
**Sex**		
Data available	1475	100.0
Female	612	41.5
Male	863	58.5
**Treatment Category**		
Data available	1475	100.0
New	1220	82.7
Retreatment (Relapse)	225	15.3
Retreatment (Failure)	17	1.2
Lost to follow up	13	0.9
**Disease Classification**		
Data available	1475	100.0
Pulmonary	1288	87.3
Extra-pulmonary	187	12.7
**Sputum Microscopy**		
Data available	1475	100
Negative	322	21.8
Positive	745	50.5
Not done	408	27.7
**HIV Status**		
Data available	1475	100
HIV Negative	644	43.7
HIV Positive	728	49.4
HIV status unknown	103	7.0

A total of 728 (49.4%) were HIV positive, while 644 (43.7%) were HIV negative. The rest had an unknown HIV status. Table 2 shows the baseline characteristics stratified by HIV status. Females accounted for 46.0% and 37.6% of HIV positive and negative patients respectively. A total of 613 (84.2%) of HIV positive patients had pulmonary compared to 584 (90.7%) of HIV negative patients. Sputum microscopy was positive in 295 (40.5%) and 397 (61.6%) of HIV positive and HIV negative patients respectively. Antiretroviral therapy (ART) data were available for 480 (65.9%) of the HIV positive patients. Of these, 411(85.6%) were on HIV treatment at TB diagnosis.

**Table 2:** baseline characteristics of tuberculosis notification in Kweneng West, Botswana, 2008 to 2016 by HIV status (N=1475)

Variable	HIV positive (n= 728)		HIV negative (n= 644)		HIV unknown (N=103)	
	Number	%	Number	%	Number	%
**Age (Years)**						
Data available	728	100.0	644	100.0	103	100.0
Median (IQR)	37 (30-46)		36 (25-54)		36 (27-54)	
Age range (years)	17-88		17-93		17-99	
**Age Category**						
16-25	84	11.5	173	26.9	22	21.4
26-35	249	34.2	147	22.8	29	28.2
36-45	207	28.4	103	16.0	22	21.4
46-55	101	13.9	69	10.7	7	6.8
56-65	57	7.8	72	11.2	8	7.8
>65	30	4.1	80	12.4	15	14.6
**Sex**						
Data available	728	100.0	644	100.0	103	100.0
Female	335	46.0	242	37.6	35	34.0
Male	393	54.0	402	62.4	68	66.0
**Treatment Category**						
Data available	728	100.0	644	100.0	103	100.0
New	605	83.1	528	82.0	87	84.5
Retreatment (Relapse)	112	15.4	102	15.8	11	10.7
Retreatment (Failure)	8	1.1	7	1.1	2	1.9
Lost to follow up	3	0.4	7	1.1	3	2.9
**Disease Classification**						
Data available	728	100.0	644	100.0	103	100.0
Pulmonary	613	84.2	584	90.7	91	88.3
Extra-pulmonary	115	15.8	60	9.3	12	11.7
**Sputum Microscopy**						
Data available	728	100.0	644	100.0	103	100.0
Negative	188	25.8	116	18.0	18	17.5
Positive	295	40.5	397	61.6	53	51.5
Not done	245	33.7	131	20.3	32	31.1
**HAART Treatment**						
Data available	480	65.9				
Not on HAART	69	14.4				
On HAART	411	85.6				

Table 3 shows the outcomes of the entire cohort of 1475 TB cases. Of these 849 (57.6%) completed treatment while 358 (24.3%) were cured, resulting in a treatment success rate of 81.9%. A total of 138 (9.4%) died during TB treatment, while 16 (1.1%) failed treatment. The rest were either lost to follow up (2.7%) or were transferred out (5.0%). The treatment outcomes are stratified by HIV status in Table 4. Among HIV positive patients, 455 (62.5%) completed treatment, while 130 (17.9%) were cured. The mortality rate was 11.4%. Among the HIV negative patients, 347 (53.9%) completed treatment, while 204 (31.7%) were cured. The mortality rate was 5.3% in this cohort. TB patients with an unknown HIV status had a mortality rate of 20.4%. The treatment completion rate and cure rate were 45.6% and 23.3% respectively in this cohort.

**Table 3:** tuberculosis treatment outcomes in Kweneng West, Botswana, 2008 to 2016 (N=1475)

Outcome	Number	%
Data available	1475	100
Completed	849	57.6
Cured	358	24.3
Lost to follow up	40	2.7
Died	138	9.4
Failed	16	1.1
Transferred out	74	5.0

**Table 4:** tuberculosis treatment outcomes in Kweneng West, Botswana, 2008 to 2016 stratified by HIV status (N=1475)

	HIV Positive		HIV Negative		HIV Status Unknown	
Outcome	Number	%	Number	%	Number	%
Data available	728	100	644	100	103	100
Completed	455	62.5	347	53.9	47	45.6
Cured	130	17.9	204	31.7	24	23.3
Lost to follow up	16	2.2	16	2.5	8	7.8
Died	83	11.4	34	5.3	21	20.4
Failed	7	1.0	9	1.4	0	0
Transferred out	37	5.1	34	5.3	3	2.9

Table 5 shows the predictors of mortality in the total cohort of 1475 patients. Compared to the 16-25 years age group, all age categories were associated with higher mortality. The >65 years age group was the most strongly associated with mortality (AOR 9.63). Male sex was also associated with mortality (AOR 1.63). Patients with positive microscopy were significantly less likely to die than patients with negative microscopy (AOR 0.50). However, patients with no microscopy performed were more likely to die (AOR 1.77). Finally, both HIV positive patients (AOR 2.13) and patients with an unknown HIV status (AOR 4.47) were more likely to die during TB treatment than HIV negative patients.

**Table 5:** predictors of tuberculosis mortality in Kweneng West, Botswana, 2008-16 (N=1475)

Variable	Crude Odds Ratio	95% CI	p value	Adjusted Odds Ratio	95% CI	p value
**Age**						
16-25	Reference			Reference		
26-35	5.25	2.21-12.50	<0.001	4.24	1.75-10.30	0.001
36-45	3.87	1.57-9.53	0.003	2.78	1.10-7.04	0.031
46-55	6.46	2.56-16.27	<0.001	5.36	2.07-13.89	0.001
56-65	5.59	2.12-14.76	0.001	4.35	1.60-11.83	0.004
>65	11.38	4.53-28.54	<0.001	9.63	3.71-24.95	<0.001
**Male Sex**	1.76	1.21-2.58	0.004	1.63	1.09-2.43	0.016
**Treatment Category**						
New patient	Reference			Reference		
Re-treatment for relapse	1.04	0.64-1.68	0.869	0.94	0.57-1.57	0.818
Retreatment for failure	0.60	0.08-4.57	0.622	0.66	0.08-5.28	0.697
**Disease Classification**						
Pulmonary TB	Reference			Reference		
Extra-pulmonary TB	1.11	0.67-1.85	0.686	0.56	0.32-0.97	0.039
**Sputum Microscopy**						
Negative Microscopy	Reference			Reference		
Positive Microscopy	0.43	0.27-0.69	0.001	0.50	0.30-0.82	0.007
Microscopy not performed	1.58	1.02-2.54	0.040	1.77	1.12-2.81	0.015
**HIV Status**						
HIV Negative	Reference			Reference		
HIV Positive	2.31	1.53	<0.001	2.13	1.35-3.34	0.001
HIV status Unknown	4.60	2.55	<0.001	4.47	2.38-8.40	<0.001

## Discussion

We report on the 9-year outcomes of DS-TB patients in a rural district in Botswana. There were more male than female patients. This was expected as globally, TB is known to affect more males than females due to reasons such as health seeking behaviour, smoking and working conditions [2]. Previous studies have had similar findings [3, 6]. In the study by Shastri *et al*. two thirds of TB patients were males [6]. In a study in Botswana, males accounted for 56% of all TB patients [3]. Almost 50% of the TB patients were HIV positive while 7% had an unknown HIV status. This means 93% of patients knew their HIV status. This finding is similar to what has been previously reported in Botswana [3]. While this is encouraging, interventions are needed to ensure that almost all people know their HIV status.

Similar to previous studies, there were significant differences in treatment outcomes between HIV positive and HIV negative patients [6, 11-14]. Favourable outcomes of treatment completion and cure were experienced by 849 (57.^6^%) and 358 (24.3%) of the cases. This means that treatment success rate was 81.9%. This treatment success rate is still below the Botswana National TB Program target of 85%, emphasizing the need to increase efforts to initiate patients and ensure that they complete treatment. Patients with an unknown HIV status had the worst outcomes since their completion and cure rates were only 45.6% and 23.3% respectively resulting in a success rate of 68.9%. Patients with an unknown HIV status may have poor health seeking behaviour including declining the HIV testing on TB diagnosis or before, seeking care late and not adhering to treatment. Future studies should explore these factors further. This study underlines the need for availability of confirmed TB diagnosis, HIV counselling and testing, adherence to ART, close follow-up and monitoring of this cohort at facility level. At district and national level, public health interventions are needed to address the above-mentioned barriers to services.

The treatment success rates (TSR) were higher than what was reported for an Indian province (2010-2011). In this cross-sectional study, the TSR was 75% in HIV positive patients and was better in people who were on ART. Similar to our study, the TSR were not significantly different from the HIV negative cohort [6]. However, the TSR in our study were lower than in an Ethiopian study. In this study, the overall success rate was very high at 91.5% and was 88.2% and 93.6% for HIV positive and HIV negative patients respectively. However, the investigators reported lower cure rates than our study [11]. In neighbouring South Africa, TSR was 75.5% among HIV positive patients and 84.7% among HIV negative patients. Thus, the unfavourable outcome rates were 24.5% and 15.3% for HIV positive and HIV negative patients respectively [12].

As for the unfavourable outcomes, both treatment failure and loss of follow-up rates were low. These findings are consistent with previous research in Sub-Saharan Africa. In an Ethiopian cross-sectional study, the failure rate was 1.2% while the default rate was 2.6% [11]. In a South Africa, there were no reports of treatment failure, while 11% of patients were lost to follow up [13]. In another South African study, 10.45% of participants were lost to follow up. The rates of these unfavourable outcomes were generally lower in our study. While this is encouraging, sustained efforts and close follow up of patients is still needed to maintain or even improve TB patient outcomes in Botswana.

The TB mortality rate in Kweneng West was 9.4% and was highest in patients with an unknown HIV status. The mortality rate was lower than what was reported in Mahalapye district, Botswana. In this cross-sectional study of TB mortality between 2013 and 2015, the overall TB mortality was 11.4% and was 13.6% among HIV positive patients [3]. Given the high loss to follow up of 10.5% in the Mahalapye study, it is possible that the difference in mortality rates could be even higher. The differences in mortality rates between Kweneng West and Mahalapye could be due to differences in HIV prevalence. The HIV prevalence in Kweneng West is among the lowest in the country [9]. Lower mortality rates on TB treatment have been reported in Kayelitsha, South Africa [15]. This could be because of a stronger healthcare system in South Africa.

Multiple independent predictors of mortality were identified in this analysis. These should inform public health policies in order to meet the Sustainable Development Goals and the National End TB Strategy targets. HIV positive patients and patients with an unknown HIV status were more likely to die during TB treatment than HIV negative patients. This finding is consistent with multiple other studies [15-18]. In order to reduce TB mortality in Botswana, interventions should also target reducing the incidence of HIV, especially advanced HIV. This must include coordination of the HIV and TB programs. Male sex was significantly associated with mortality in our cohort. Investigators in Mahalapye had a similar finding [3]. However, female sex was significantly associated with mortality [15]. Our study underlines the need to design interventions that target men. Men tend to have worse health seeking behaviour than women and often present late for care. They may have lower CD4 counts and other comorbidities. Unfortunately, these factors were not evaluated in our study due to the limitations of data. Future prospective studies should include these variables and other possible confounders. Generally, advancing age was associated with higher mortality, which was highest in patients aged more than 65 years. This finding is consistent with previous studies [3, 15, 16]. Older patients often have multiple comorbidities which increase their risk of death.

TB patients with a positive microscopy were significantly more likely to die during TB treatment than those with negative microscopy. In contrast, patients with no microscopy performed were more likely to die than patients with negative microscopy. Similar studies in Africa have had similar results [15, 17, 18]. Patients with a positive microscopy have a definitive diagnosis and can be promptly started on appropriate treatment and they might show a better adherence to the anti TB treatment (ATT) knowing that their disease has been clearly and accurately identified and is curable. In contrast, patients with a negative microscopy often have multiple investigations before TB diagnosis is made and, culturally, since the lab has “failed” to name the cause of their sufferings, they might resist subsequent tests such as HIV testing. This delays treatment initiation. In addition, patients with negative microscopy may have alternative diagnoses.

Surprisingly, patients with extra-pulmonary TB (EPTB) were less likely to die during TB treatment than patients with pulmonary TB (PTB). Future prospective studies should explore this further. EPTB has been shown to be associated with higher mortality in multiple studies. In an Ethiopian study EPTB patients were 17 times more likely to die that PTB patients [19]. In South Africa, the risk of death was 2.7 times. In another Ethiopian study, the risk of death was 2.7 times in EPTB patients. EPTB is associated with advanced HIV disease. Furthermore, some EPTB sites including TB meningitis have very high mortality [19, 20].

**Limitations:** this study was not without limitations. Firstly, this was a retrospective study using programmatic data. It was therefore inevitable that there would be missing data and some inaccuracies in reporting. The results of this study could also be affected by bias and confounding. Selection bias was limited by including all TB patients in the district during the study period. Confounding was managed by stratified analysis and multiple logistic regression analysis. However, the multivariate analysis was limited to the variables that are routinely collected and reported to the BNTP. Other possible confounders including severity of the disease and presence of comorbidities were not captured. Despite the limitations, this is a very critical study that should inform policy both at district and national level.

## Conclusion

The favourable treatment outcomes of success and cure were generally high. The outcomes were better for HIV negative patients and were worst for patients with an unknown HIV status. Similarly, the mortality was highest among patients with an unknown HIV status. Multiple predictors of mortality were identified. Public health interventions should target these risk factors. These should include HIV testing and early linkage to care and initiation of therapy. At national and district level, TB and HIV programs should work closely together if the burden and outcomes of TB in Botswana are to be improved.

### What is known about this topic

- Tuberculosis is common in settings of high HIV prevalence;
- Tuberculosis outcomes are often poor in resource limited settings;
- HIV positive patients have worse TB outcomes than HIV negative patients.

### What this study adds

- The TB success rates in a rural Botswana district are comparable to other settings in the region;
- The TB mortality rates in the district are high and are associated with multiple risk factors including HIV status, advancing age, sputum microscopy results and male sex.
